# Embedding nutrition into routine oncology care: about time?

**DOI:** 10.3389/or.2026.1639494

**Published:** 2026-03-20

**Authors:** Barry J. A. Laird, Lorenzo Antonuzzo, Darío Sánchez Cabrero, Ece Esin, Fieke Froeling, Karin Jordan, Domina Kekez, Richard Skipworth, Marieke Schooneman, Şuayib Yalçın, Carla M. Prado, Maurizio Muscaritoli

**Affiliations:** 1 Edinburgh Cancer Research Centre, University of Edinburgh, Edinburgh, United Kingdom; 2 St Columba’s Hospice, Edinburgh, United Kingdom; 3 University of Florence, Florence, Italy; 4 La Paz University Hospital, Madrid, Spain; 5 Ankara Medipol University, Department of Internal Medicine, Ankara, Türkiye; 6 University of Glasgow, Glasgow, United Kingdom; 7 Department of Hematology, Oncology and Palliative Medicine, Ernst von Bergmann Hospital Potsdam, Potsdam, Germany; 8 Department of Hematology, Oncology and Rheumatology, University Hospital Heidelberg, Heidelberg, Germany; 9 University Hospital Zagreb, Zagreb, Croatia; 10 Clinical Surgery, University of Edinburgh, Royal Infirmary of Edinburgh, Edinburgh, United Kingdom; 11 University of Edinburgh, Edinburgh, United Kingdom; 12 Academic Medical Center, University of Amsterdam, Amsterdam, Netherlands; 13 Department of Medical Oncology, University Institute of Cancer, Ankara, Türkiye; 14 Department of Agricultural, Food and Nutritional Sciences, Faculty of Agricultural, Life and Environmental Sciences, University of Alberta, Edmonton, AB, Canada; 15 Department of Translational and Precision Medicine, Sapienza University of Rome, Rome, Italy

**Keywords:** cancer, nutrition, oncology, staging, palliative, cachexia

## Abstract

**Background:**

Cancer significantly impacts nutritional status, thereby affecting treatment tolerance, quality of life, and survival. However, nutrition remains underutilized in oncology care.

**Methods:**

This paper describes current evidence to examine challenges, and propose strategies, for embedding nutrition into routine oncology care across the cancer continuum.

**Results:**

The complexity of cancer-related malnutrition demands simple, validated, and cancer-specific tools for nutritional screening and assessment. Terminology such as “cachexia” and “malnutrition” may not appear positive for clinicians or patients; alternatives like “nutritional fitness” are suggested. Barriers to integration include misconceptions about cachexia as solely end-of-life, poor adherence to clinical guidelines, limited training for oncologists, and lack of access to dietitians. Integration of nutrition into clinical drug trials and perioperative care (e.g., prehabilitation) is essential. Emerging tools like PRONTO and composite indices incorporating body composition and inflammation may offer practical pathways forward.

**Conclusion:**

Early and ongoing nutritional monitoring, positive communication, regulatory alignment, and education of all stakeholders, including oncologists, surgeons, and patients are critical to embedding nutrition in oncology. By reframing nutritional care as a core component of treatment rather than supportive care, outcomes can be improved and practice re-shaped.

## Background

Cancer is the second leading cause of death worldwide, representing one of the greatest health challenges with around one in five people being diagnosed with cancer during their lifetime (1). Malnutrition is highly prevalent among patients with cancer (2), with rates varying depending on cancer type, stage, and assessment criteria. Studies suggest studies show that at across all cancer types at least 30% of patients have malnutrition (3). The negative effects of malnutrition involve all the touchpoints and stages of the patient journey, affecting quality of life, treatment options (including intensity of treatment, tolerance and side-effects) and prognosis (4–7). Notably, malnutrition is directly responsible for up to 10%–20% of cancer deaths (8).

Optimising nutritional status is now advocated as being a central tenet of cancer care (9). When early detection of malnutrition and risk factors are done, followed by classification, then tailored educational and treatment strategies, then this has the potential to improve outcomes. Evidence from randomized clinical trials is limited and heterogeneous, but efforts should be made to implement evidenced-based guidelines (e.g., European Society for Clinical Nutrition and Metabolism [ESPEN] guidelines) to offer recommendations for patient management in cancer multidisciplinary teams (4–6, 10–12).

Overall, comprehensive nutritional intervention(s) may improve quality of life and, in certain circumstances, oncological outcomes. However, there is an urgent need to separate, the pharmaconutrients, pharmacological agents, behavioral and medical interventions that have proven efficacy against malnutrition (5, 6). Furthermore, there is accumulating evidence supporting the need to identify special categories of patients in whom nutritional intervention should be targeted, such as patients undergoing curative or palliative surgery, those with high risk of severe malnutrition (e.g., chemoradiotherapy for upper gastrointestinal or head and neck cancers, high-dose chemotherapy, hematopoietic stem-cell transplantation), patients with cognitive impairments, geriatrics, and cancer survivors (13–15).

**FIGURE 1:**
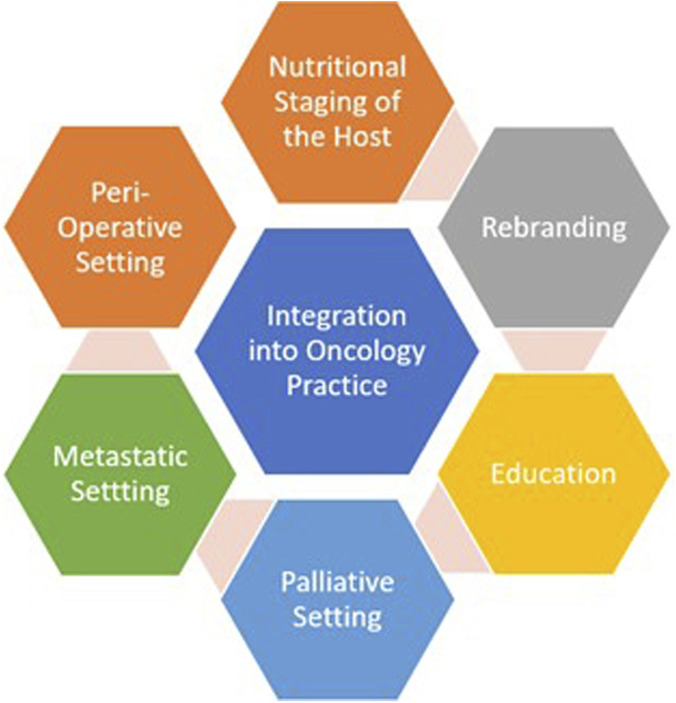
Strategic pillars for advancing nutrition in oncology.

In this article, we highlight the role of nutrition in improving outcomes across cancer care, examine the current barriers preventing its integration into oncology practice, and propose strategies spanning communication, education, research, and policy, to embed nutrition as a core component in oncology practice (Figure 1). As a narrative review, this work does not follow a systematic search methodology; rather, references were selected based on the collective expertise of the authors, seminal contributions in the field, and studies most relevant to the themes discussed.

## Stage the tumour, nutritionally stage the patient

In oncology, significant emphasis is placed on cancer staging, including radiological, genetic, and biological assessments, which are critical for guiding treatment decisions. However, this focus on tumour-specific factors often overlooks the patient’s overall condition, particularly their nutritional status. This oversight neglects a key determinant of treatment tolerance, response, and overall outcomes.

While tools like the Eastern Cooperative Oncology Group Performance Status (ECOG-PS) are routinely used to evaluate physical function, they fail to account for nutritional parameters, despite physical function being a key component of comprehensive nutritional assessment. Poor nutritional status, including declines in physical function, not only negatively affect the effectiveness of cancer therapies but also negatively impacts quality of life (QOL) and is associated with worse prognostic outcomes (16).

This is particularly pronounced in patients in highly catabolic states. For them, the heightened metabolic demands imposed by anticancer treatments and the disease itself can exacerbate nutritional deficits, further compounding their vulnerability. Low muscle mass and sarcopenia may also be present, representing poor nutritional status that negatively impact treatment tolerance and outcomes. Addressing these challenges requires a more comprehensive approach to patient assessment, integrating nutritional evaluation as a standard component of oncology care to optimise treatment outcomes and improve overall patient wellbeing (17, 18).

Therefore, individualised staging and a subsequent nutrition care plan is essential to counter malnutrition in patients with cancer. Screening and then assessment of nutritional status is important to identify patients who are at risk of malnutrition. If a patient is screened as being at risk, a comprehensive diagnostic assessment should be undertaken. So far, there is no general agreement on the ‘best’ screening tool; however, the Malnutrition Universal Screening Tool (MUST), the Nutrition Risk Screening 2002 (NRS-2002), the Short Nutritional Assessment Questionnaire (SNAQ) or the Malnutrition Screening Tool (MST) are valid options. Patients who are at risk of malnutrition should be re-screened at regular intervals, e.g., every 3 months. Recently, the evidence-based PROtocol for NuTritional risk in Oncology (PRONTO) was proposed to simply and rapidly identify oncology patients at risk of malnutrition, thereby facilitating their assessment and follow-up by the multidisciplinary team (19). Through three simple questions, PRONTO explores three domains (namely, body weight loss, low appetite/food intake and reduced muscle strength) which are independent predictors of negative outcomes in cancer patients. Validation of this quick nutritional risk screening tool for oncologists is needed and currently ongoing.

Regarding nutritional assessment following positive screening, no single standard method is recommended. ESMO’s cachexia guideline recommend objective assessment of patients’ nutritional and metabolic status (including weight, weight loss, body composition, inflammatory state (20, 21), nutritional intake and physical activity) and examination for the presence of factors interfering with the maintenance or improvement of this status, including nutrition impact symptoms, GI dysfunction, chronic pain and psychosocial distress (22).

One important but often overlooked aspect in clinical practice is that patients who ‘do not look malnourished’, (particularly those overweight or with obesity), may still have significant nutritional deficits. Their body weight can mask underlying muscle wasting or inadequate intake, leading clinicians to underestimate their nutritional risk. Since malnutrition in individuals with excess body weight is not recognized though visual assessment alone, this underscores the need for accurate and objective nutritional screening and assessment, then to identify at-risk individuals and guide interventions.

## Nutritional care in the peri-operative and neo-adjuvant setting

Cancer-associated malnutrition is often highly prevalent even in those undergoing surgery for localised disease with radical intent. In patients with high-risk cancer types in higher income countries, rates of pre-operative weight loss can be as high as 35% (23). Importantly, such pre-operative weight loss is highly prognostic and associated with worsened patient outcomes. In a large, multinational study using the Global Leadership Initiative on Malnutrition (GLIM) criteria, severe malnutrition was present in 33·3% of patients with colorectal or gastric cancer undergoing surgery, with a disproportionate burden in upper-middle-income countries (44·4%) and low-income and lower-middle-income countries (62·5%) (24). Critically, after adjustment for patient and hospital risk factors, severe malnutrition was associated with an increased risk of 30-day mortality across all country income groups (high income: adjusted odds ratio [aOR] 1·96 [95% CI 1·14–3·37]; upper-middle income: 3·05 [1·45–6·42]; low income and lower-middle income: 11·57 [5·87–22·80]), demonstrating that severe malnutrition mediated an estimated 32% of early deaths in low-income and lower-middle-income countries and an estimated 40% of early deaths in upper-middle-income countries (24).

Beyond weight loss alone, in recent years, there has been significant attention on computerized tomography (CT) pre-operative body composition analysis for patient prognostication. Such analyses have tended to focus on assessments of muscularity, but measures of adiposity are also available (25). Other direct and indirect approaches to assess body composition in clinical settings, facilitate the identification of patients with or at risk for malnutrition (26), and the tracking of effectiveness interventional strategies (27). Furthermore, such assessments can be combined with markers of systemic inflammation to generate novel scores with the potential for greater prognostic accuracy. For example, in surgical patients with locally advanced oesophagogastric malignancy, the cachexia index (calculated using the CT skeletal muscle index, serum albumin and neutrophil-lymphocyte ratio) was associated with disease progression during neoadjuvant treatment, worsened postoperative mortality, and shortened overall survival (28). The benefits of such composite scores are that they may be especially useful when considering treatment options in patients with borderline resectable disease. However, despite the major impact of malnutrition and cachexia on surgical patients prognoses, nutritional parameters and body composition are rarely considered in a systematised fashion in multi-disciplinary team decision-making. There is a clear and unmet need to provide robust and repeated nutritional assessment during neoadjuvant treatment and the perioperative setting. In recent consensus studies of clinicians caring for surgical patients with oesophagogastric malignancy, it has been agreed that nutrition should play a key component of any multimodal prehabilitation programme (29). Such prehabilitation is recommended to improve response to neoadjuvant therapy; improve chemotherapy completion rates; optimise the body composition of patients undergoing neoadjuvant chemotherapy; improve muscle strength; optimise physical fitness and postoperative quality of life (QoL); reduce surgical complication rates; and shorten postoperative length of stay (29). Exactly how such nutritional support should be delivered, however, is less clear. In recent studies, individualised nutritional interventions have been shown to improve post-operative chemotherapy tolerance and QoL, and reduced adverse effects in patients with colorectal cancer. Furthermore, there is persistent interest in the role of immunonutrition and specialised amino-acid combinations to reduce surgical complications.

## Nutritional care in systemic treatment for incurable cancer

Due to advances in therapeutic options for patients with metastatic cancers, metastatic disease has transitioned into a chronic condition in recent years. Depending on the specific cancer site, patients with metastatic cancer can live for many years with oncological treatment. However, these individuals often experience a range of symptoms from the disease itself, as well as side effects from the treatment (30).

Cachexia is a complex syndrome characterised by hormonal and metabolic imbalances in the early stages, evolving into energy metabolism disorders and protein catabolism issues as the disease progresses (8). Additionally, cachexia and fatty infiltration of muscle tissue are recognised as risk factors that can diminish chemotherapy tolerance in these patients. The development of cachexia is gradual, making early detection of risk factors crucial for mitigating treatment complications through timely nutritional support. Given the high catabolic state of these patients, it is essential to consider their increased caloric and protein requirements. The recommended daily energy intake for these patients ranges from 25–30 kcal/kg, along with approximately 1.5 g/kg of protein. This raises the question of whether these needs can be met through a regular high-calorie diet, or if oral nutritional supplements (ONS) are necessary (31). ONS can help achieve higher caloric and protein intake in smaller volumes, utilising calorie- and protein-dense formulas which can significantly increase macro- and micro-nutrient intakes. When ONS are not sufficient to meet the nutritional needs, then enteral nutrition (EN) should be considered.

Early identification of risk of cachexia and prompt initiation of EN can lead to improved energy intake, reduced cachexia risk, enhanced quality of life, decreased chemotherapy side effects, and increased treatment efficacy (32). Given these facts, the early initiation of enteral nutrition should be regarded as an integral component of the therapeutic regimen for these patients, alongside their specific oncological treatments. Notably, initiating nutritional support early, well before refractory cachexia develops, allows time for interventions to be effective, before the window of reversibility closes (33).

## Nutritional care in the palliative setting

One of the challenges with the term “palliative” is that it can often be mistaken for end-of-life care; yet patients can be under palliative care for months and indeed years with an incurable cancer. Therefore, it is important that the stage of the patient is taken into account as the needs of those who have a prognosis of many months, will differ from the needs of patients in the last weeks of life (5).

Incorporating nutritional support into the palliative setting offers significant potential to enhance the quality of life (QoL) for patients facing advanced cancer (34). Physical activity, tailored to individual abilities, can help maintain muscle mass, improve mobility, and support emotional wellbeing, even in late-stage care. Coupled with nutrition support, these interventions can help reduce fatigue, stabilise weight, and provide comfort, ensuring patients maintain dignity and a sense of control during their final stages of life.

Pain management is a critical but often underexplored aspect of nutrition-related care. Malnutrition, dehydration, or metabolic imbalances can exacerbate pain or other discomforts. Providing adequate nutrition can mitigate these issues, complementing pharmacological pain management strategies (35). Dietitians and healthcare providers can collaborate to create tailored nutritional plans that address pain triggers while supporting overall wellbeing (36).

According to clinical guidelines, in some palliative care situations, the decision to initiate enteral or parenteral nutrition should be based on a careful evaluation of prognosis, goals of care, and the patient’s overall condition (37). For individuals with a life expectancy of several months or more, individualized nutritional support, including artificial nutrition when appropriate, can help maintain weight, reduce treatment-related side effects, and preserve quality of life (34). Enteral or parenteral nutrition may be beneficial even in patients with poor performance status if aligned with their care goals. However, in the final weeks of life, with substantial metabolic disturbances and inflammation, the benefit of artificial nutrition becomes limited and may increase discomfort. In such cases, the focus should shift to comfort, symptom management, and psychosocial support. Regular reassessment and shared decision-making are essential to ensure nutrition interventions remain aligned with patient-centered outcomes.

Despite these benefits, palliative nutritional support is frequently constrained by financial limitations, such as insufficient reimbursement and high out-of-pocket costs. These barriers can prevent patients from accessing necessary resources, including specialised diets, supplements, or enteral nutrition. Advocacy for policy changes to improve funding and reimbursement for palliative nutrition care is essential. By addressing these limitations, healthcare systems can better integrate comprehensive, patient-centred approaches to enhance QoL and ensure equitable access to end-of-life nutritional support.

## Overview of targeted nutrition interventions

Nutrition intervention plays an essential role and oral nutritional supplements (ONS) remain a cornerstone strategy, particularly when regular food intake is insufficient. When initiated early and maintained consistently, it can help prevent the ‘wildfire’ of the rapid catabolic response that many patients experience during the cancer trajectory (38).

Individualized nutritional support can substantially impact patient outcomes. A secondary analysis from the EFFORT trial, demonstrated that such support during hospitalization reduced 30-day mortality by more than 5%, improved functional outcomes, and enhanced quality of life in patients with various types of cancer, independent of cancer type or treatment status. This real-world, pragmatic intervention emphasized a tiered strategy using dietitian-guided plans, food fortification, ONS, and escalation to enteral or parenteral support when needed. Together, this growing body of evidence reinforces that nutritional care is not an adjunct, but a critical, life-saving component of comprehensive cancer treatment (39).

A variety of nutritional components have shown promise in supporting patient’s nutritional status, including those focusing on the management of low muscle mass in cancer (39). Adequate protein intake, often exceeding 1.2 g/kg/day, is fundamental to support muscle protein synthesis, particularly during periods of active treatment (40). Specific amino acids, such as leucine, play a key role in stimulating muscle-building pathways, and their metabolite, β-hydroxy β-methylbutyrate (HMB), has been shown to reduce muscle protein breakdown and may be especially beneficial in patients unable to meet protein targets (41). Other nutrients like creatine can improve muscle strength and functional outcomes, while omega-3 fatty acids, particularly eicosapentaenoic acid (EPA), have anti-inflammatory properties that may help counteract muscle wasting. Vitamin D also supports muscle function and is frequently deficient in cancer populations. Emerging evidence suggests that multi-nutrient strategies, combining protein, HMB, EPA, and vitamin D, may offer synergistic benefits in preserving or restoring muscle mass in individuals with cancer (9).

Ongoing clinical trials in the field of sarcopenia and cachexia are primarily focused on a targeted set of nutritional interventions. According to a scoping review, the most commonly studied strategies include ONS, omega-3 fatty acids (particularly EPA), protein-rich products, and HMB, either alone or in combination with n-3 fatty acids (42). Nutrition counselling is also frequently integrated as part of a combined intervention approach. These strategies aim to preserve or enhance muscle mass, strength, and physical performance in patients with cancer, especially those who are older adults.

## The importance of nutrition education: What is relevant and when?

Educational programmes for medical oncologists should embrace a more integrated and holistic approach to patient-centered care, extending beyond a narrow focus on nutritional support (43). Effective education should encompass comprehensive strategies for managing drug toxicity, offering practical guidance on recognising and mitigating adverse effects, and administering enteral or parenteral nutrition when necessary (44). Furthermore, training should address symptom management techniques for nausea, vomiting, pain, and other common treatment-related challenges, ensuring oncologists are well-equipped to support patients through every stage of their care (45).

A key focus should be placed on the broader role of nutrition in cancer treatment (46). This includes its influence on drug metabolism and efficacy, the relationship between nutritional status and treatment tolerance, and the critical importance of maintaining muscle metabolism for recovery and overall outcomes. Such technical content would engage oncologists by aligning with their interest in evidence-based and treatment-focused education.

Currently, nutrition is often overlooked not only in medical schools (47) but also in oncology training, frequently regarded as outside the scope of their responsibilities. This perception limits its integration into clinical practice and undermines its potential to enhance patient outcomes. By embedding nutrition education within the broader context of oncology care and emphasizing its direct impact on treatment success, programming can reshape these attitudes. Empowering oncologists with the knowledge and tools to incorporate nutritional considerations into their practice will foster a more comprehensive, patient-centered approach, ultimately improving both the quality and efficacy of cancer care.

## Does nutrition in oncology need rebranded?

The terminology surrounding malnutrition and cachexia in oncology care is often perceived as negative and stigmatising, potentially discouraging engagement from both patients and healthcare providers (48). To address this, alternative terms such as metabolic health, metabolic wellbeing, nutritional wellbeing, nutritional balance, and nutritional advocacy have been proposed. These terms convey a more positive and empowering message, emphasising proactive health management rather than focusing solely on deficits or illness (49).

Integrating nutritional screening and assessment into drug trials and oncology studies is also a vital step toward embedding nutrition into cancer care (50, 51). Framing nutrition as a tool to enhance treatment response through positive messaging, such as promoting metabolic health or advocating for nutritional wellbeing, researchers and clinicians can help shift perceptions away from seeing nutrition as merely supportive. Embedding nutritional endpoints into trials not only strengthens the scientific evidence base but also signals the importance of nutrition to regulatory bodies, clinicians, and patients alike. For example, in studies utilising CT scans to evaluate body composition, particularly muscle mass, can provide valuable insights into patients’ nutritional and physical health as part of comprehensive care. This dual-purpose approach allows for a more holistic understanding of treatment outcomes and reinforces the relevance of nutritional assessment in modern cancer care.

In the rehabilitation setting, fostering a message of “nutritional fitness” alongside regular physical activity can promote a holistic approach to recovery and long-term health (52). This messaging not only underscores the importance of maintaining optimal nutritional status but also highlights the role of physical fitness in improving treatment outcomes, reducing recurrence risks, and enhancing quality of life. By combining these strategies with a focus on empowerment and positive messaging, this approach can improve patient engagement and contribute to better outcomes across the cancer care continuum.

Equally important is the use of appropriate language to shape perceptions and experiences in oncology. Using person-first terminology, such as “patients with cancer” instead of “cancer patients”, emphasizes the individual rather than the disease (53). This subtle but important change in terminology reinforces the idea that a person is not defined by their diagnosis and aligns with broader efforts to humanize cancer care.

## Is it time to incorporate nutrition into oncology guidelines?

Despite the growing evidence highlighting the impact of malnutrition on cancer outcomes, a major difficulty encountered in clinical practice is the frequent omission of nutritional management in most specific guidelines for various cancer types. With a few exceptions (like general advice for nutritional care included within ESMO´s cachexia guidelines (22)) the majority of recommendations are found in specialised nutrition guidelines, like those issued by ESPEN (5). This approach presents several challenges. While the latter provide clear and detailed recommendations for screening and overall patient management, these can often be too broad and not entirely practical for an effective implementation by non-specialised clinicians. As a result, their endorsement by oncologists tends to be inconsistent, leading to frequent underutilization (54, 55), despite their acknowledged importance (43).

Particularly, there is a notable deficiency in nutritional recommendations within the primary oncology guidelines for high-risk cancer patients, such as head and neck or upper gastrointestinal cancers. For these particular tumors, numerous studies have proven the significant benefits of specific nutritional interventions (56), especially in the perioperative setting (57) and during radiotherapy (58). However, to date, there have been no precise recommendations for nutritional screening or management at any stage of these cancers. As the current general guidelines are hardly applicable for these singular tumors, it remains a crucial task to incorporate individual nutritional approaches into their oncology guidelines.

It is time to redefine nutritional management in oncology. It may be insufficient for both Oncology and Nutrition societies to elaborate their own recommendations; rather, a novel multidisciplinary approach requires integrating the directives from nutrition societies into oncology guidelines as part of an optimal supportive oncology plan. Since cancer is a heterogeneous pathology, nutritional recommendations may need to be more tumor-specific rather than broadly generalized. To ensure clinical relevance and effectiveness, this evolving understanding should be reflected in oncology guidelines through evidence-based, tailored nutritional strategies. Achieving meaningful change will require broad stakeholder engagement. Involving not only oncologists and dietitians but other healthcare professionals, and patient advocacy groups is essential to building the momentum needed to embed nutrition into every stage of cancer care.

## Conclusion

A comprehensive approach to nutritional care in oncology is essential to improving patient outcomes, quality of life, and treatment efficacy. Despite the well-documented impact of malnutrition on cancer prognosis, nutritional management remains underutilised in clinical practice. Early screening, individualised assessment, and timely interventions are crucial for optimising patient care. Integration of nutritional guidelines into oncology protocols, alongside enhanced education for oncologists, will help bridge existing gaps with key tips listed in Table 1. Future research should focus on refining evidence-based interventions and ensuring equitable access to nutritional support. By prioritising nutrition, oncology care can become more holistic, ultimately benefiting both patients and healthcare systems.

**TABLE 1:** Top ten tips in oncology nutritional care.

Tip	Comment
Early and continuous nutritional monitoring	Nutritional screening and evaluation should be initiated at the time of cancer diagnosis and continued throughout the cancer journey
Complexity of cancer nutrition assessment	Always consider multiple factors when assessing nutritional status, including muscle mass, inflammation, cachexia, and treatment-related nutritional effects
Carefully assess food intake	Anorexia and food intake should be differentially explored and intake matched with nutrient needs in any phase of the disease
Need for simple and validated tools	Use and support the adoption of simple, evidence-based nutrition screening and assessment tools and pay special attention to body composition, especially changes in muscle mass, as a key element of nutritional evaluation
Use more patient-friendly terminology	Patients are frequently confused by terms like anorexia, malnutrition, cachexia, and this generates alarm and decreases awareness of the relevant body composition changes occurring with cancer
Use positive terms in any type of communication	The concept of “nutritional fitness” should be promoted and consolidated in both scientific and patient communication
Improve communication and collaboration between oncology and nutrition	Cachexia is still mistakenly considered an end-of-life condition, and nutrition a palliative measure. This frequently prevents timely-appropriate nutritional prevention and therapy in cancer patients
Integrate nutrition in research	Nutrition research gains greater attention when integrated with clinical drug trials, enhancing the likelihood of positive oncological outcomes. Cancer nutrition studies should include oncology relevant outcomes besides nutritional ones
Implement guideline application	Clinical practice shows a generally low adherence to guidelines on nutritional support and palliative care
Beat barriers to nutritional therapy	Consistent nutritional care faces challenges, including a lack of awareness among oncologists and insufficient support staff such as dietitians. The concept of nutritional prehabilitation prior to medical or surgical treatment should be implemented and consolidated
